# Multi-layer topological transmissions of spoof surface plasmon polaritons

**DOI:** 10.1038/srep22702

**Published:** 2016-03-04

**Authors:** Bai Cao Pan, Jie Zhao, Zhen Liao, Hao Chi Zhang, Tie Jun Cui

**Affiliations:** 1 State Key Laboratory of Millimetre Waves, School of Information Science and Engineering, Southeast University, Nanjing 210096, China; 2 Synergetic Innovation Center of Wireless Communication Technology, Southeast University, Nanjing, 210096, China; 3 Cooperative Innovation Centre of Terahertz Science, No. 4, Section 2, North Jianshe Road, Chengdu 610054, China

## Abstract

Spoof surface plasmon polaritons (SPPs) in microwave frequency provide a high field confinement in subwavelength scale and low-loss and flexible transmissions, which have been widely used in novel transmission waveguides and functional devices. To play more important roles in modern integrated circuits and systems, it is necessary and helpful for the SPP modes to propagate among different layers of devices and chips. Owing to the highly confined property and organized near-field distribution, we show that the spoof SPPs could be easily transmitted from one layer into another layer via metallic holes and arc-shaped transitions. Such designs are suitable for both the ultrathin and flexible single-strip SPP waveguide and double-strip SPP waveguide for active SPP devices. Numerical simulations and experimental results demonstrate the broadband and high-efficiency multi-layer topological transmissions with controllable absorption that is related to the superposition area of corrugated metallic strips. The transmission coefficient of single-strip SPP waveguide is no worse than −0.8 dB within frequency band from 2.67 GHz to 10.2 GHz while the transmission of double-strip SPP waveguide keeps above −1 dB within frequency band from 2.26 GHz to 11.8 GHz. The proposed method will enhance the realizations of highly complicated plasmonic integrated circuits.

## Introduction

Surface plasmon polaritons (SPPs) are propagating surface modes that are excited on the interface of metal and dielectrics with their normal components of electric fields decaying exponentially in near-infrared and visible frequencies^1^. Such surface modes show great confinement and much shorter operating wavelength properties, providing a brand new approach to overcome the diffraction limits^2^,^3^, miniaturize the photonic components, and build highly integrated optical components and circuits. In the past decades, SPPs have been studied in a variety of applications such as high-resolution imaging^4^,^5^, electromagnetically induced transparency^6^,^7^,^8^, photovoltaic improvement^9^,^10^, and biosensing^11^,^12^,^13^.

However, such surface modes with significant properties cannot be excited at the lower frequency band, since metal shows a perfect electric conductor (PEC) property in the microwave or lower-terahertz frequencies. In order to take advantages of SPP modes, a series of metamaterial structures have been presented to mimic the SPP-like waveguides. In 2004, Pendry *et al*. proposed a metamaterial design with periodic cubic holes perforated on the metal surface to achieve the first spoof SPP waveguide^14^. The calculated dispersion results show that the surface mode excited by the vertical illumination has an SPP-like property. Since then, many researches on supporting and transmitting spoof SPP waves have been reported^15^,^16^,^17^,^18^,^19^,^20^,^21^. Spoof SPP modes have been designed and experimentally observed in both terahertz and microwave frequencies in 2008 and 2009^15^,^16^. Then a spoof SPP waveguide was proposed using two rectangular waveguides as the excitation of a domino-like transmission line^17^.

Recently, an ultrathin conformal SPP design has been reported using corrugated metallic strip^18^, which provides long-distance transmission with small loss and shows good ability of model shaping due to the flexible substrate. In 2014, a highly efficient, broadband, and ultrathin spoof SPP waveguide was proposed^19^, which contains the momentum matching parts with gradually altering grooves to excite and receive SPP modes efficiently. Later, other types of SPP-propagation designs have been presented to generate the surface modes on corrugated and their complementary structures by microstrip lines^20^,^21^. Such SPP waveguides provide perfectly broadband transmission properties with low loss, showing great potentials in the application of plasmonic devices and circuits.

Based on such highly efficient designs, a series of device applications have been achieved such as the narrow-band and broadband SPP filters using the metamaterial particles^22^, the mixed circuit of SPP and localized surface plasmon particle^23^, and the space radiators with radiation directions controllable^24^. More recently, a new spoof SPP waveguide structure with two antiparallel ultrathin corrugated strips has been proposed, which is convenient to integrate with active chips to produce active SPP devices (e.g. the power amplifiers^25^ and mixers). The double-strip SPP waveguide also demonstrated higher confinements and less cross-talk properties compared with the traditional transmission lines, breaking the challenges in signal-integrity problem emerged in integrated circuits and systems^26^.

Despite the fact that spoof SPPs have been used in a series of devices, the application of such surface modes is mainly limited on basic passive and active components. In order to take advantages of spoof SPPs in complicated plasmonic circuits and systems, here we propose the topological transmissions of SPP modes from one layer to the other layer using metallic holes and arc-shaped transitions, connecting different parts of both ultrathin single- and double-strip SPP waveguides. More importantly, the additional overlapping unit cell of the corrugated structures close to the connecting hole could provide a power rejection without disturbing the rest transmissions. The rejection frequency is mainly decided by the area of unit cell. The proposed method is easily realized and could provide efficient transmissions between different layers.

### Multi-layer SPP transmissions on single-strip waveguides

**Figure 1:**
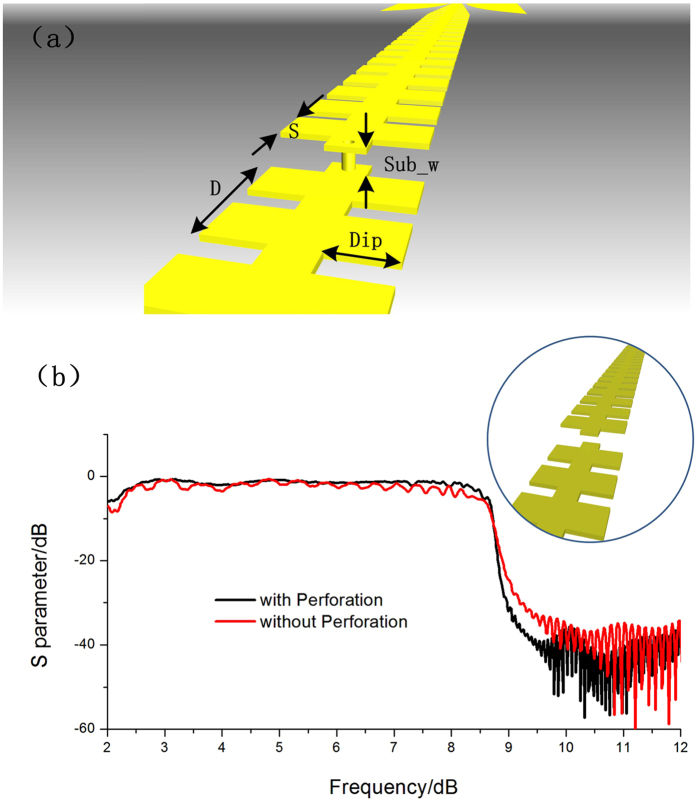
(**a**) Schematic structure of the multi-layer transition for two ultrathin single-strip SPP waveguides on different layers, in which Dip, D, and S are the depth, cycle, and gap between adjacent grooves, respectively, and Sub_w is the thickness of the substrate. (**b**) The transmission coefficients of the two-layer single-strip SPP waveguides with (the black line) and without (the red line) the metallic hole, in which the insert map is the structure of dual-layer SPP waveguides without the metallic hole.

The schematic structure of the multi-layer transition of two ultrathin single-strip corrugated waveguides is illustrated in Fig. 1(a), in which the substrate is hidden for clear view. The design contains two SPP waveguides in different medium layers, whose sides are connected by a metallic hole with the radius of 0.3 mm as transition. As was fully discussed in Ma’s work^19^, such corrugated waveguides could provide highly-efficient and broadband low-pass transmissions of SPP modes without using the traditional metallic background. The cutoff property of the waveguide is mainly decided by the dimensions of the periodic grooves and substrate. The smaller the depth of grooves is, the higher the cutoff would be. And the smaller and thinner the relative permittivity and width of the substrate are, the higher the cut-off frequency would be. Here we choose the commercially available printed circuit board (F4B) as the substrate, whose relative permittivity is 2.65, loss tangent is 0.001, and thickness (Sub_w) is 0.5 mm. The depth, cycle, and gap between adjacent grooves are Dip = 4 mm, D = 6 mm, and S = 4 mm, respectively, which lead to a cutoff frequency 10.2 GHz for the ultrathin single-strip SPP waveguide.

The metallic hole is mainly used to form the surface mode on the transition part, instead of the waveguide mode caused by the boundaries of two metallic layers. The comparison of transmission coefficients between the multi-layer SPP waveguides with and without the metallic hole is illustrated in Fig. 1(b), in which the thickness of the substrate is 0.8 mm. From this figure, we clearly observe that, the transmission efficiency of the SPP waveguide with the perforation (the black line) is up to 2 dB higher than that of the waveguide without the perforation (the red line). Meanwhile, the cutoff effect is much better for the case with metallic hole, in which a more cliffy falling edge is observed. When the thickness thickens, the different of transmission efficiencies between designs with and without metallic hole would increase, since it would be harder to couple signal from one side of substrate to another without perforation.

**Figure 2:**
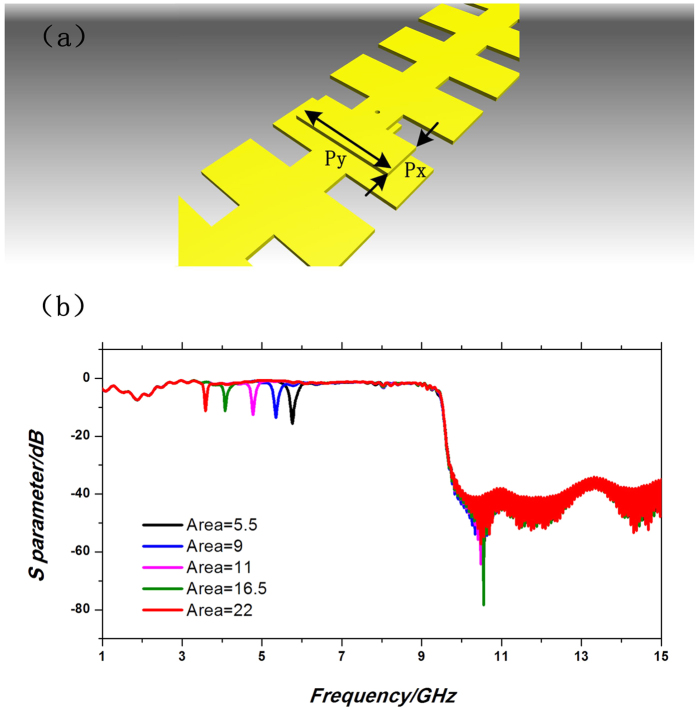
(**a**) Schematic structure of the multi-layer transition of two single-strip SPP waveguides with the overlapping patch. (**b**) Transmission coefficients of the SPP waveguides with different overlapping areas.

If the transition metallic hole is perforated at the raised part instead of the concave part of the SPP waveguide, or an extra unit of groove overlaps the other part of the waveguide, an extra strong resonant mode will be excited. Such a resonance is mainly caused by the field oscillation between the overlapping parts of two waveguides, and the resonant frequency is determined by the overlapping area. The design with an overlapping patch is exhibited in Fig. 2(a) as example. The size of the additional patch is described by its length 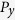 and width 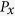, as shown in Fig. 2(a). The simulated transmission results with different overlapping areas are demonstrated in Fig. 2(b), in which we find that a series of high-quality resonances over −18 dB appear, while the transmissions in the other propagating bands keep high performance. The resonant frequency has red shift as the area increases. By modulating the size of overlapping rectangular patch, we could conveniently control the absorption point and provide a tunable rejection property to the basic SPP waveguide.

**Figure 3:**
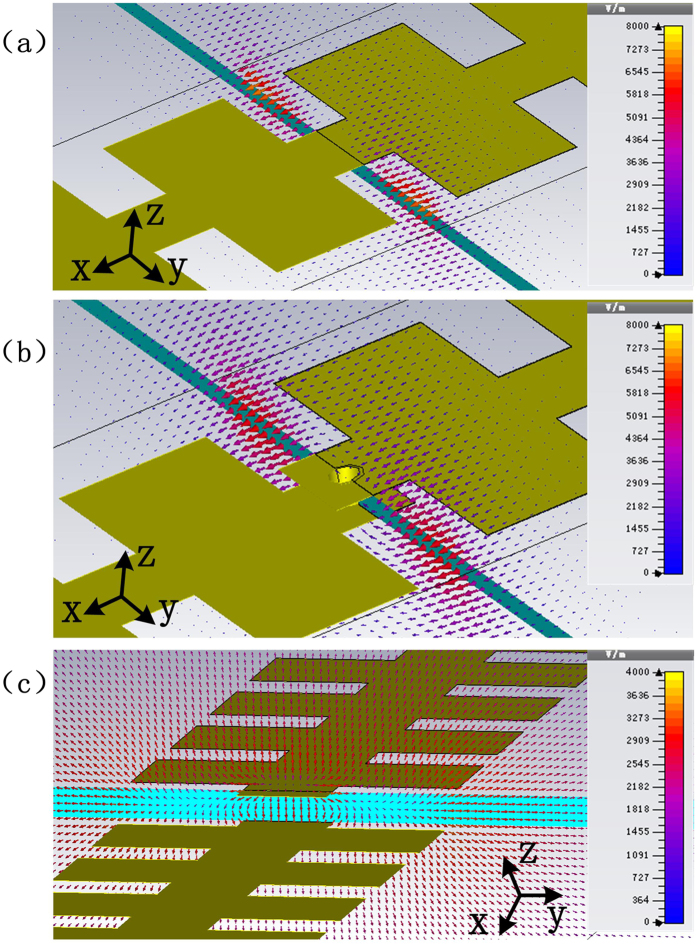
Near-electric-field distributions of (**a**) the normal ultrathin single-strip SPP waveguide, (**b**) the multi-layer transition of two SPP waveguides with perforation of metallic hole, and (**c**) the multi-layer transition of two SPP waveguides without perforation of metallic hole.

In order to further understand the role of the metallic hole and the mode transition during the multi-layer transmission of SPP modes, the near-field distributions around the ultrathin SPP waveguides and the transition parts with and without the metallic hole are presented in Fig. 3. Here, the high confinement property of SPPs can be expressed by their near fields, which are mainly oscillated inside and near the periodic grooves (see Fig. 3(a)). We note that the field inside the groove oscillates along the *x* direction and decays exponentially with the distance away from the waveguide. When the SPP wave needs to propagate through the medium to the other side of substrate, the center of oscillation alters from the top layer to the bottom layer, and this would bring in unexpected mode aberration and radiation loss. The metallic hole on the transition may help keep the surface mode and the unique properties. Figure 3(b) gives the near fields in the transition part of the dual-layer SPP waveguides, which keep oscillating inside the groove along the *x* direction while the confinement property may decrease slightly. For the waveguide without perforation, however, the transmission is mainly based on the field coupling, and the surface mode would be destroyed due to the electric boundaries of the SPP waveguides on two layers. As shown in Fig. 3(c), the field strength is significantly weakened according to the color bars, and the field distribution is clearly no longer highly confined near the groove.

### Multi-layer topological SPP transmissions on double-strip waveguides

**Figure 4:**
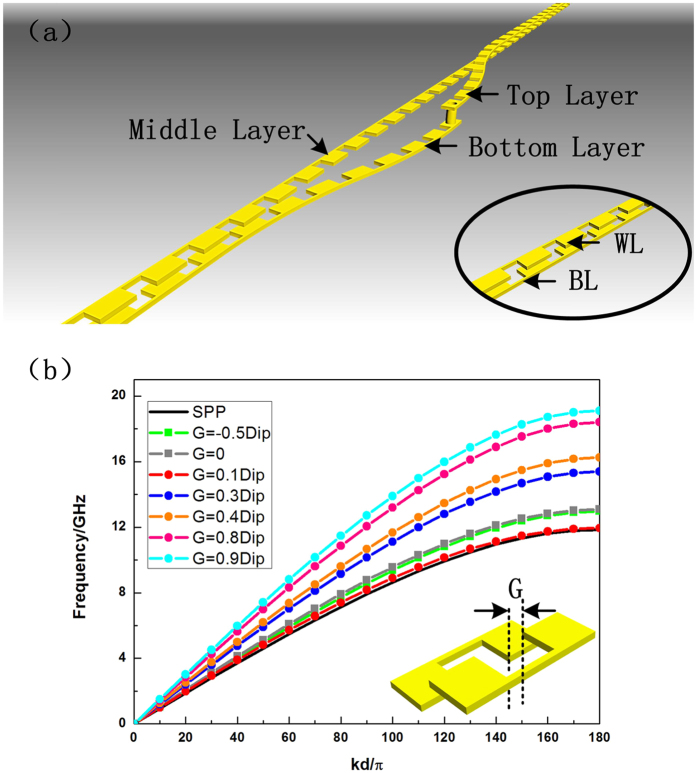
(**a**) Schematic structure of the multi-layer topological transition for two double-strip SPP waveguides. The insert map in the lower right corner is the basic structure of the normal double- strip SPP waveguide. (**b**) The dispersion relations under different distances G in the horizontal direction.

For active SPP devices and circuits, the ultrathin single-strip SPP waveguides are not convenient to integrate with amplifier chips in the microwave frequency. Therefore, a complex design with two anti-symmetrical ultrathin corrugated metallic strips, the double-strip SPP waveguide, was introduced^25^, as shown in the insert map in the lower right corner of Fig. 4(a). The two anti-symmetrical corrugated strips are named as the waveguide layer and the background layer, separately, for convenience. For the multi-layer SPP transmissions of two double-strip waveguides, the transition part is much more complicated than that of the single-strip design, which contains an extra arc-shaped topological transition for connecting the two double-strip SPP waveguides. As illustrated in Fig. 4(a), the background layer is set at the middle medium layer, while the two parts of the waveguide layers on the top and bottom medium layers are connected by two arc-shaped topological transition lines and a metallic hole. What also distinguishes double-stripe design from that on single-strip design is that the effects of substrates are not the same. The smaller and thicker the relative permittivity and width of the substrate are, the higher the cutoff frequency of double-strip design would be. Such a design is also convenient for the situation that both waveguide and background layers are required to change their positions to other medium layers or the forms of waveguide on both sides of transition part are not the same. The momentum matching could also be realized according to the analysis in Fig. 4(b). Under such circumstances, the change of grooves’ depths in transition part needs to be introduced and the transition part and is no longer symmetric.

The dispersion properties of the transition part are exhibited in Fig. 4(b), in which the inset shows the basic unit model. Here, G and Dip represent the distance between two parts of the unit in the horizontal direction and the groove depth, respectively. By modulating the magnitude of G, such a unit model could be described as the basic model of the double-strip SPP waveguide. The black line in the figure indicates the dispersive property of the SPP waveguide with the relationship


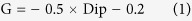


when it comes to the arc-shaped topological transmission line, with G gradually increasing from 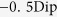 to 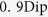, the frequency point of cutoff property shows a rise-and-fall destabilization, and then shifts to a higher frequency. The balanced point is determined when the distance G meets the following demand


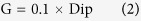


The red line with dots in the figure shows that the SPP unit under the situation of Eq. (2), which has a similar form to the complementary spoof SPPs, has nearly the same dispersion property as the normal double-strip SPP structure. This gives a good fundamental for the arc-shaped transmission line to be the transition part, avoiding the serious mode-mismatching loss.

**Figure 5:**
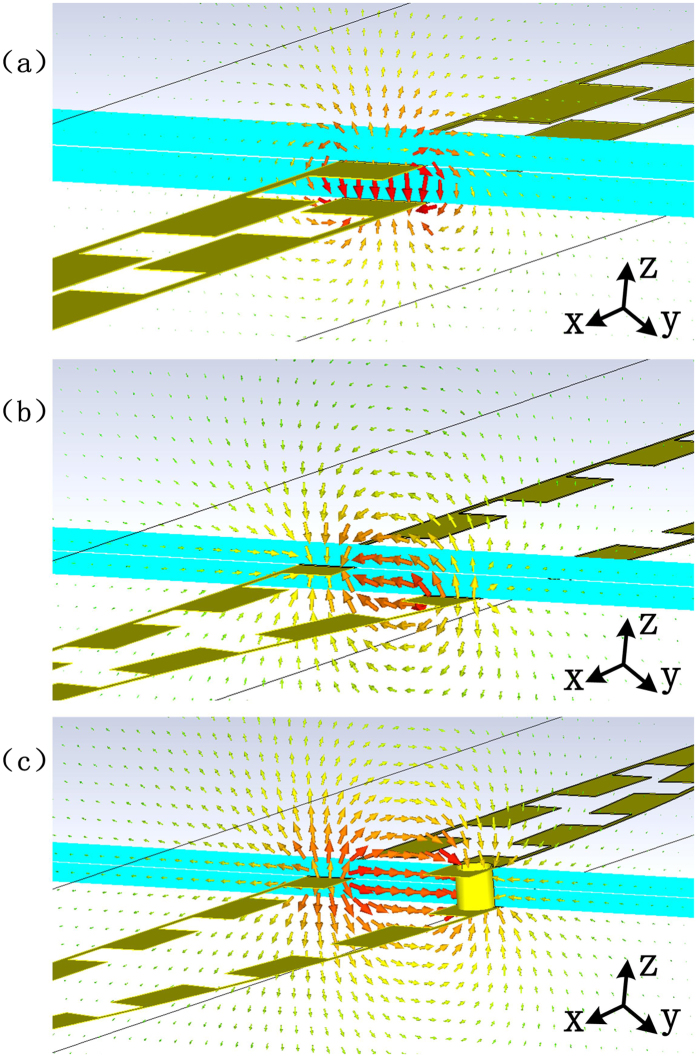
The near electric field distributions of (**a**) the normal double-strip SPP waveguide, (**b**) the arc-shaped topological transition area for multi-layer SPP transmission, and (**c**) the metallic hole area for multi-layer SPP transmission of double-strip SPP waveguides.

To make direct exhibition of mode transfer during the transition, we examine the near-field distributions of the double-strip SPP waveguide. The basic SPP mode excited inside the waveguide would oscillate between the waveguide and background layers, as illustrated in Fig. 5(a). We notice that the electric fields are mainly confined inside the waveguide. In the topological arc transition part, the oscillation direction of the surface mode alters, as shown in Fig. 5(b). But it is still highly confined between two corrugated metallic structures, and the distribution of surface mode is just like that of complementary structures^21^. At the perforation point, the metallic hole helps keep the electric field oscillating between the waveguide layer and background layer, as displayed in Fig. 5(c), avoiding the extra loss of mode hybridization caused by the resonance between two parts of the waveguide layer.

### Experiments and results

**Figure 6:**
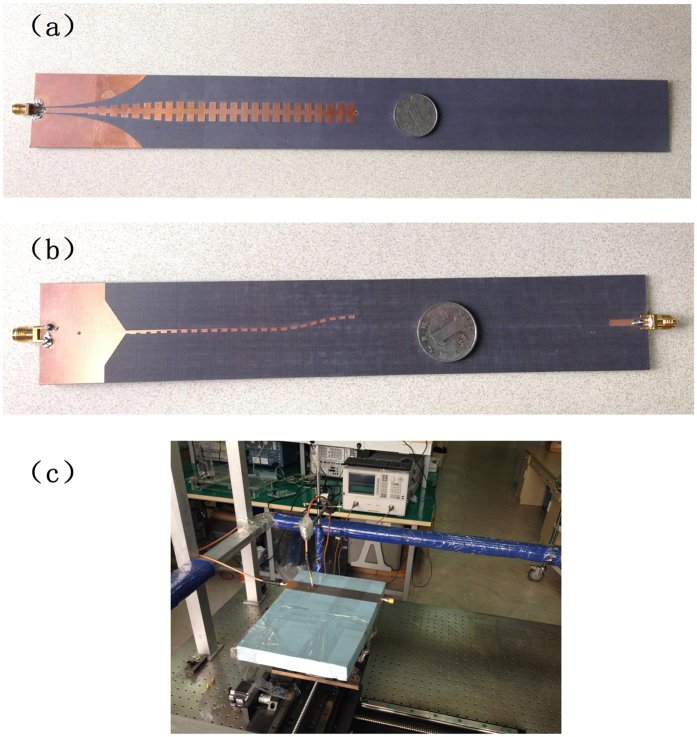
The fabricated samples of the multi-layer SPP waveguides. (**a**) The single-strip SPP waveguide. (**b**) The double-strip SPP waveguide. (**c**) Photo of the two-dimension near field scanner.

**Figure 7:**
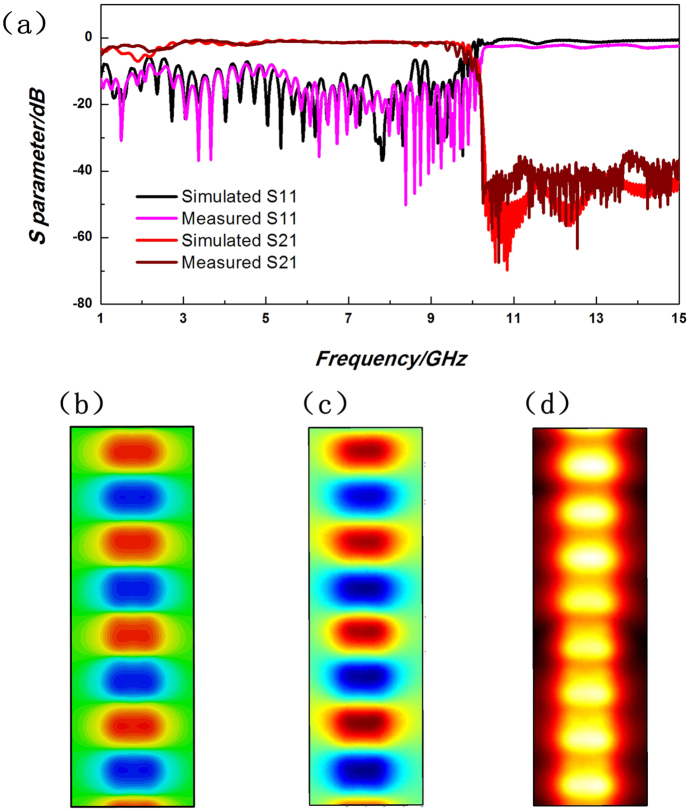
The comparison of simulated and measured results for the multi-layer single-strip SPP waveguides. (**a**) The SPP transmission and reflection coefficients. (**b**) The simulated and (**c**) the measured results of transient electric field distributions (relative values) at 8 GHz. (**d**) The measured magnitude distributions of electric fields (relative values) at 8 GHz.

**Figure 8:**
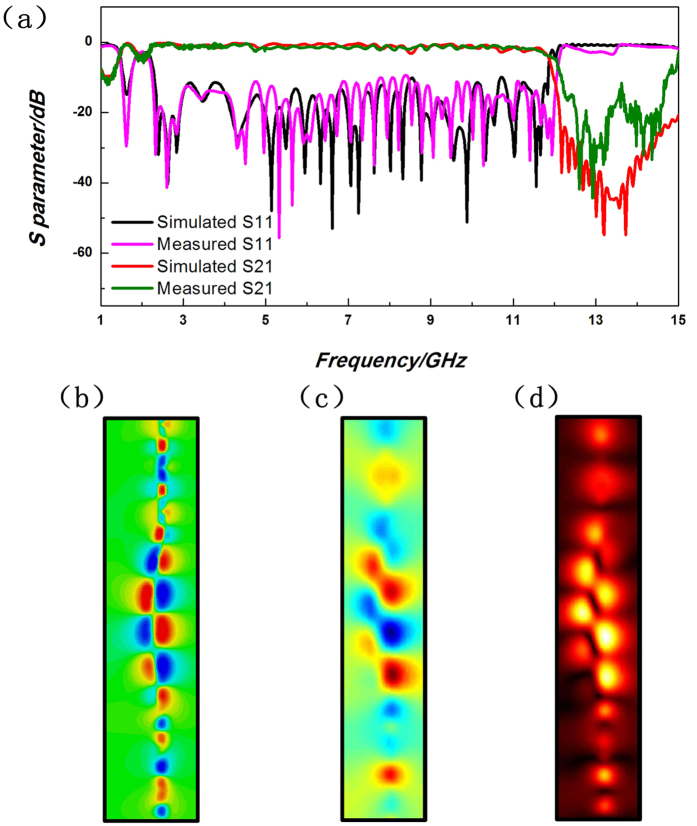
The comparison of simulated and measured results for the multi-layer double-strip SPP waveguides. (**a**) The SPP transmission and reflection coefficients. (**b**) The simulated and (**c**) the measured results of transient electric field distributions (relative values) at 10 GHz. (**d**) The measured magnitude distributions of electric fields (relative values) at 10 GHz.

We have fabricated two samples of the multi-layer topological transitions for both the single- and double-strip ultrathin SPP waveguides (see Fig. 6), and measured their transmission/reflection properties and near electric fields. Near electric distribution is measured by the scanner consisting of a position controllable plate, a field-detection probe and an Agilent Vector Network Analyzer shown in Fig. 6(c). Two 50 Ω coaxial cables are used connecting the Vector Network Analyzer with the sample and the vertical probe as excitation and receiver. The platform moves along both directions step by step and the near electric field distribution is plotted by reading the measured data via MATLAB. Figures 7 and 8 demonstrate comparisons of the SPP transmission coefficients and the near-field distributions between numerical simulations and experimental results for the double-layer single- and double-strip SPP waveguides, respectively.

In Fig. 7(a), we clearly observe that the ultrathin single-strip SPP waveguide with perforation of metallic hole could provide a nearly perfect transmission in a broad frequency band, just like the normal single-strip SPP waveguide behaves. The measured scattering parameters (the pink line for the reflection coefficient S11 and the wine red line for the transmission coefficient S21) agree excellently with the numerical simulations (the black line for S11 and the red line for S21), with the transmission level as high as −0.8 dB within frequency band from 2.67 GHz to 10.2 GHz. Figure 7(b,c) give the transient electric filed distributions at 8 GHz, in which the SPP modes propagate from the top layer to the bottom layer, and the metallic-hole transition is located at the middle point of the figure. The measured results in Fig. 7(c) are highly consistent with the simulated results in Fig. 7(b). The magnitude distribution of the electric filed is given in Fig. 7(d), from which we notice a clear exhibition that the energy-distribution area has been decreased when the energy is transmitted into the bottom layer via the metallic hole, because of the highly confinement of SPP modes.

For the case of double-strip SPP waveguide, the SPP transmission and reflection properties are measured in Fig. 8(a), in comparison with the simulation results. It is obvious that the measured values are almost the same as the simulations except that the cutoff frequency has a blue shift of 0.27 GHz, because of the deviation in the processing technique of multi-layer printed circuit board and the unsteadiness of its relative permittivity. The transmission keeps above −1dB within frequency band from 2.26 GHz to 11.8 GHz. The distributions of simulated and measured transient electric fields and their magnitudes at 10 GHz are illustrated in Fig. 8(b–d). Since the near fields of the SPP modes on double-strip configuration are mainly confined inside the structure, it is inconvenient to measure the near-field distributions inside. Hence, the measured results are slightly weaker than numerical simulations, and the magnitudes around the arc-shaped topological transition part are stronger than those near the SPP waveguide.

## Conclusion

The multi-layer topological transmission of electronic signals is a basic requirement in the modern integrated circuit technologies, and it is a very important issue for the plasmonic integrated circuits when used in engineering applications or combined with the traditional communication systems. Here, we have proposed a general method to realize highly efficient transmissions of SPP waves between different medium layers for both single-strip and double-strip ultrathin SPP waveguides. We can also achieve a controllable absorption property by adding an overlapping corrugated unit on the transition part. The metallic hole we perforated across the substrate of the SPP design helps keep the confinements of the exited surface modes and increase the transmission efficiency. Such designs have great potential in the multi-layer plasmonic integrated circuits and systems.

## Methods

Numerical simulations of the single- and double-strip ultrathin SPP waveguides were performed by the commercial software, CST Microwave Studio. The substrate printed with the SPP structures was a commercial printed circuit board (F4B) with the relative permittivity 2.65 and loss tangent 0.001. In experiments, we used the Agilent vector network analyzer to measure the SPP transmission and reflection coefficients, and used a home-made near-field scanner to measure the electric fields of the fabricated samples through a small probe.

## Additional Information

**How to cite this article**: Pan, B. C. *et al*. Multi-layer topological transmissions of spoof surface plasmon polaritons. *Sci. Rep.* **6**, 22702; doi: 10.1038/srep22702 (2016).
